# Epi-fingerprinting and epi-interventions for improved crop production and food quality

**DOI:** 10.3389/fpls.2015.00397

**Published:** 2015-06-05

**Authors:** Carlos M. Rodríguez López, Mike J. Wilkinson

**Affiliations:** Plant Research Centre, School of Agriculture, Food and Wine, Faculty of Sciences, University of AdelaideAdelaide, SA, Australia

**Keywords:** Fingerprinting, epigenetics, crop biotechnology, crop plants, crop quality, crop protection, crop improvement, priming

## Abstract

Increasing crop production at a time of rapid climate change represents the greatest challenge facing contemporary agricultural research. Our understanding of the genetic control of yield derives from controlled field experiments designed to minimize environmental variance. In spite of these efforts there is substantial residual variability among plants attributable to Genotype × Environment interactions. Recent advances in the field of epigenetics have revealed a plethora of gene control mechanisms that could account for much of this unassigned variation. These systems act as a regulatory interface between the perception of the environment and associated alterations in gene expression. Direct intervention of epigenetic control systems hold the enticing promise of creating new sources of variability that could enhance crop performance. Equally, understanding the relationship between various epigenetic states and responses of the crop to specific aspects of the growing environment (epigenetic fingerprinting) could allow for a more tailored approach to plant agronomy. In this review, we explore the many ways in which epigenetic interventions and epigenetic fingerprinting can be deployed for the improvement of crop production and quality.

## Context

The sustained growth in food production over the 50 years since the start of the green revolution can be at least partly explained by the introduction of molecular approaches to crop breeding (Evenson and Gollin, 2000). Systematic marker-assisted introgression has now become a mainstay of genetic improvement programs (Collard and Mackill, 2008) and yet some of the most successful varieties of several crops have arisen spontaneously, and have been identified by simple phenotypic selection. These so-called ‘sports’ are far more common in crops that are propagated vegetatively, and can often form a substantial proportion of the varieties grown. The source of the observed phenotypic divergence in sports is often assumed to have a genetic rather than epigenetic origin (Schmitz et al., 2013). In either case, the genetic divergence between sports and their progenitor lines is inevitably minimal, and so are notoriously difficult to differentiate using conventional molecular markers (Breto et al., 2001). The reality is that for the vast majority of instances we do not fully understand how phenotypic variability can be explained at the molecular level (Ball, 2013). This uncertainty is often exacerbated by poor trait definition and a lack of genomic resolution (King et al., 2010) but may sometimes arise from a mistaken presumption of genetic rather than epigenetic causality (Breto et al., 2001; Rois et al., 2013). Ever since Waddington (1942) first proposed the term epigenotype to describe the interface between genotype and phenotype, the science of epigenetics has been progressively adding more layers of complexity to our knowledge of how information is stored and utilized within the living cell. Recent years has seen a dramatic increase in the depth of understanding of how epigenetic control mechanisms operate. There is now growing desire to better understand the stability and role of epigenetic regulatory systems in controlling development, shaping the phenotype, and determining the physiological resilience of higher organisms surviving in fluctuating environments (Geyer et al., 2011; Bräutigam et al., 2013).

Epigenetic processes can affect a phenotype without altering the genetic code (Bird, 2007) and can operate in a number of ways to alter the availability or efficacy of DNA sequences for transcription; determine transcript identity or amend the longevity of mRNA transcripts in the cell (for review, see Chahwan et al., 2011) or by changing the stability or activity of protein products. The many epigenetic mechanisms that mediate these effects include modifications of histone tags, ATP-dependent chromatin remodeling, polycomb/trithorax protein complexes, chemical modification on DNA bases and regulatory processes directing mRNA degradation and alterations to DNA chemistry driven by small RNA molecules, with circular RNA as the latest addition (Wilusz and Sharp, 2013) to the many small RNAs that fulfill this role (i.e., lncRNA, siRNA, microRNA). This array of processes is clearly interconnected and almost certainly acts in a complex, interactive and redundant fashion (Grant-Downton and Dickinson, 2005; Berger, 2007). Describing all the methods developed to study all the mentioned epigenetic layers is outside the scope of this review and we will instead focus on the potential role of the best-studied epigenetic mechanism, DNA methylation, as a route to elicit new advances in crop improvement.

## Epigenetic Interventions and Crop Improvement

Applied epigenetics is an area of science that is evolving rapidly and spawning new opportunities for the enhancement of crop production. DNA methylation involves the addition of a methyl group to carbon 5 of cytosine bases (forming 5-methylcytosine, 5mC). In plants, DNA methylation can occur in three contexts (i.e., CG, CHG, or CHH, *H* = a nucleotide other than G). DNA methylation occurring within promoters or coding regions typically act to repress gene transcription. RNA-directed DNA Methylation (RdDM) is an important mechanism by which plants can achieve targeted DNA methylation to reduce expression of a particular gene (Wassenegger et al., 1994). This form of gene silencing is directed by small interfering RNAs (siRNAs) and is often associated with the silencing of transposable elements (TEs). However, the system can also repress the expression of endogenous genes, especially those positioned close to TEs. RdDM relies on the activity of DICER-like 3 (DCL3), Argonaute 4 (AGO4) and the DNA-dependent RNA polymerases Pol IV, and Pol V and the RNA-dependent polymerase RDR2. Collectively, the products of these genes direct the DOMAIN REARRANGED METHYLTRANSFERASE 2 (DRM2) protein to add methyl groups to Cytosines within the targeted region and so repress expression (Naumann et al., 2011). In this way the expression of genes that regulate development or cell metabolism can be altered (Becker and Weigel, 2012). The first and most direct means of exploiting this relationship is through the deliberate perturbation of global methylation patterns via exogenous interventions. This can be achieved in several ways. Most simply, chemical inhibitors of DNA methyltransferases such as 5-azacytidine or decitabine can be used to cause partial, genome-wide DNA demethylation (Stresemann and Lyko, 2008) and so generate new ‘epigenetic’ variants that hopefully include epi-alleles that confer desirable changes to crop phenotype. Amoah et al. (2012) used this strategy when they applied 5-azacytidine to seedlings of rapeseed (*Brassica napus*) and generated novel lines that exhibited increased seed protein content. This blind tactic for the release of new variation is perhaps most analogous to mutation breeding and relies on the screening of similarly large numbers of individuals to yield positive results. It nevertheless offers the tangible benefit of not requiring a deep understanding of the mechanisms involved.

A more directed approach to epigenetic intervention is made possible by reference to the relationship between changes in the growing environment and associated changes in methylation-driven gene expression. One system by which plants can increase their resilience to challenge by biotic or abiotic threats is by intensifying the responsiveness of their immune system after recognition of specific signals from their environment. This so-called ‘priming’ provides potentially long-lasting protection and is based on eliciting a faster and/or stronger reaction upon subsequent challenge by the same or related stressor (Conrath, 2011). The primed response is made possible by increased sensitivity of previously exposed plants to signal molecules such as b-aminobutyric acid (BABA), volatile organic compounds associated with herbivore damage or to strain-specific pathogen effectors (Pastor et al., 2013). Several studies indicate that the primed response of plants to pathogen attack is mediated through early and strong activation of immune response systems such as the Salicylic Acid (SA) pathway (Kohler et al., 2002; Jung et al., 2009) and the Jasmonic Acid pathway (Turlings and Ton, 2006; Heil and Ton, 2008). It is now becoming clear that RdDM-associated DNA methylation is sometimes implicated in the improved responsiveness of primed plants. For instance, Agorio and Vera (2007) showed that AGO4 is required for full resistance in *Arabidopsis* against *Pseudomonas syringae* and by implication RdDM-mediated methylation. Yu et al. (2013) showed that some TEs become demethylated in *Arabidopsis* following exposure to *P. syringae* and that this change is associated with restricted multiplication and vascular propagation of the pathogen. The authors inferred that the widespread demethylation of the TEs may have caused prime transcriptional activation of some defense genes. Other studies have similarly shown that manipulation of the growing environment can also evoke DNA methylation-mediated changes to the expression of genes that can influence yield, such as stomatal development (Tricker et al., 2012) or aspects of product quality such as vitamin E levels (Quadrana et al., 2014). Whatever the mechanism of operation leading to these effects, the ability to enhance the defensive capability of crop plants through the prior exposure to signal molecules or to disabled or denign pathogens has innate appeal. This prospect is most immediately tangible for clonal crops, where the effect of the conditioning treatment on methylation-mediated changes to phenotype need not pass through a filial generation. For most seed crops, however, there is the need that the induced changes to methylation status remains stable across generations for methylation-based priming to have practical utility. There is now growing evidence to suggest that at least some environmentally induced methylation marks can remain stable between generations, implying that intergenerational plant priming may also be possible.

Molinier et al. (2006) provided the first compelling evidence that environmentally induced epigenetic change can be retained over subsequent generations that were naïve to the eliciting factor. In this case, exposure to UV and flagellin (an elicitor of plant defenses) was seen to cause *Arabidopsis* to respond by increasing homologous recombination as detected by restoration of transgene function. Whilst the authors were unable to assign the effect to a particular epigenetic mechanism, they were able to demonstrate that the effect did not require presence of the transgene, was dominant, could be inherited from either parent and persisted for at least four filial generations. Boyko et al. (2007) subsequently found that progeny of tobacco mosaic virus (TMV)-infected plants show reduced methylation levels of R-gene-like genes, and enhanced resistance to different pathogens (Kathiria et al., 2010). Likewise, Slaughter et al. (2012) demonstrated that *Arabidopsis* exposed to localized infection by an avirulent strain of *P. syringae* or priming-inducing treatments with BABA produce descendants that are more resistant to *Hyaloperonospora arabidopsidis.* These and many other examples of transgenerational priming of resistance (for review, see Pastor et al., 2013) imply that it may be possible to supply the grower communities with seed lots as well as clonal cuttings that are primed to enhance tolerance to biotic or abiotic stresses. Delivery of such a service will depend on stability of the effect, ability to assure that the expected change to DNA methylation has occurred, and most importantly, that there are no yield penalties associated with the priming event itself. Certainly, Luna et al. (2012) demonstrated that whilst the asymmetric DNA methyltransferase (drm1drm2cmt3) triple mutant of *Arabidopsis* (blocked for RdDM-dependent DNA methylation function) is more resistant to biotrophic pathogens such as *H. arabidopsidis* and *P. syringae*, it is also more susceptible to the necrotrophic fungus *Alternaria brassicicola*. Thus, it is entirely plausible that some beneficial changes that are induced by priming may come at the expense of some associated detrimental features. The nature of such interactions will no doubt emerge with time and effort.

There are also more direct ways in which transgenerational stability of epi-alleles could ultimately be integrated into crop breeding efforts. In a landmark paper, Hauben et al. (2009) demonstrated that it is possible to obtain stable epigenotypes exhibiting improved energy use efficiency (an important yield determinant) through recurrent phenotypic selection of isogenic *B. napus* lines. Furthermore, crosses between these genetically identical but epigenetically divergent lines generated hybrids with a 5% yield increase on top of heterosis. Tricker et al. (2013a) for showed that environmentally induced epi-alleles associated with drought and low relative humidity tolerance can become fixed and remain stable over several generations. These observations raise the scope of targeted management of the growing environment during breeding to deliberately elicit and fix epigenetic changes responsible for control of a particular trait or developmental process. The high likelihood that genotypes will vary in their capacity to become primed or to remain stably fixed in a desired state (Daymond et al., 2011) provides scope for simultaneous genetic and epigenetic selection for (or against) aspects of plant plasticity and resilience. To our knowledge, this type of profiling has yet to be formally incorporated into commercial breeding efforts. In the following sections we therefore explore a range of specific approaches that hold promise to enhance contemporary crop improvement efforts.

At a more fundamental level, Cortijo et al. (2014) have provided an elegant illustration of how the transgenerational stability of some induced methylation marks can be usefully exploited for forward genetics efforts when they were able to construct linkage maps to describe the epigenetic basis of complex traits, so-called epiQTL analysis (Long et al., 2011; Cortijo et al., 2014). This strategy has the significant potential advantage over conventional QTL analysis by circumventing the need for functional mutational differences between parental genotypes of mapping populations used for forward genetics.

## Use of DNA Methylation as a Biomarker

**FIGURE 1:**
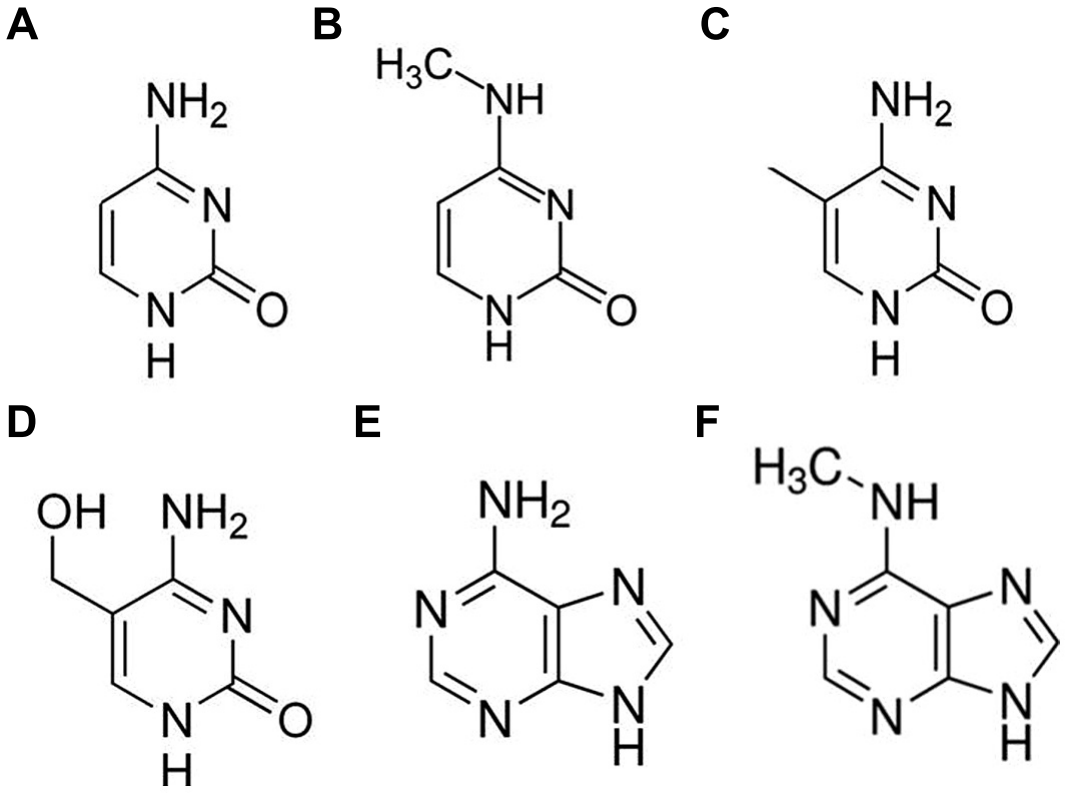
**Molecular structures of DNA bases: cytosine (A), N4-methylcytosine (m4C; B), 5-methylcytosine (5mC; C), 5-hydroxymethylcytosine (5hmC; D), adenine (E), and N6-methyladenine (m6A; F)**.

DNA methylation-dependent gene regulation plays an important role in orchestrating cellular differentiation and development (Rogers and Rogers, 1995; Manning et al., 2006; Henderson and Jacobsen, 2007; Feng et al., 2010; Ito et al., 2010; Yaish et al., 2011) and also provides the basis for genome–environment interactions that confer agility and plasticity of gene expression, and mediates molecular response to fluctuations in the living environment (Amoah et al., 2012). The genomes of almost all phyla include at least one alternate form of chemically modified base (Hattman, 2005), including N6- methyladenine (m6A), N4-methylcytosine (m4C), 5-methylcytosine (5mC), and 5-hydroxymethylcytosine (5hmC) (**Figure 1**). Of these, 5mC is by far the best studied and was originally thought to be the only functional base modification found in higher organisms (Kriaucionis and Heintz, 2009). Environmentally induced changes in 5mC have also been shown to be at least partially stable between filial generations (Tricker et al., 2013a; Cortijo et al., 2014). We are just starting to understand the mechanisms that either prevent or permit the inheritance of such epigenetic changes (Iwasaki and Paszkowski, 2014). The value of a particular 5mC as a biomarker for a particular physiological or developmental state relies partly on the consistency its association with each particular state but also on its stability. There is considerable variation in the extent to which a locus shows both consistency and stability. For example, in tomato, a spontaneous epi-allele (*cnr*) is responsible for the inhibition of fruit ripening in some epi-mutant lines (Manning et al., 2006). The methylation status of sites within this locus are highly predictive of the observed phenotype and reversions (demethylation and associated phenotypic change) occur at a frequency of roughly one in 1000. In comparison, mutability of the epigenetic silencing of the DWARF1 gene in rice occurs in around 1 in 10 plants (Miura et al., 2009). Overall, it appears that DNA methylation patterns do not fluctuate randomly between generations or in response to the environment but neither are they completely stable (Becker and Weigel, 2012). It will therefore desirable to identify specific sites or loci that are both stable and predictable for a particular state to maximize the capacity to apply epifingerprinting techniques across a wide range of germplasm and also between laboratories.

It is now emerging that other modified bases are also present in at least some eukaryotic organisms. These most notably include m6A and 5hmC, although relatively little is currently known about the distribution or function of these bases in plants (Ashapkin et al., 2002). The methylated modification of adenine, m6A, was first discovered in *Escherichia coli* and has since been found in a wide range of prokaryotes and simple eukaryotes (e.g., prokaryotes Dunn and Smith, 1955; ciliates, Hattman, 2005). In prokaryotes, it appears that m6A induces DNA conformational changes that alter protein–DNA interactions (Sternberg, 1985). There is indirect evidence that m6A may also be present in mammals (Polaczek et al., 1998; Ashapkin et al., 2002) although this has yet to be demonstrated unequivocally. There is stronger evidence for the presence of m6A in plants (Ashapkin et al., 2002), including the identification of a putative adenine DNA methyltransferase gene in the genome of *Arabidopsis thaliana* (Sternberg, 1985). While it is unclear whether m6A is essential for the regulation of eukaryotic genes, the detection of m6A residues in the DNA methylation maintenance gene DRM2 (Ashapkin et al., 2002) implies that this possibility is at least plausible, and that the presence and location of this modified base could be used for diagnostic purposes.

The alternate modification of cytosine, 5hmC, is present both in the nuclear (Kriaucionis and Heintz, 2009) and mitochondrial (Shock et al., 2011) genomes of mammals. This form of the base is far less abundant than 5mC and is typically more highly tissue-specific (Muenzel et al., 2011), perhaps implying a role in tissue differentiation and development. In plants, 5hmC has only been reported in the genome of chloroplasts (Moricová et al., 2013) although more recent publications demonstrate that it is either absent or present at undetectably low levels in plants (Erdmann et al., 2015). This form of cytosine has been proposed as an intermediate in either the active or passive demethylation of 5mC (Huang et al., 2010). However, recent evidence leads some to suggest that it may have an important functional role in its own right, at least in animals (Robertson et al., 2011a; Wu et al., 2011). Moreover, under high resolution melting (HRM) conditions 5mC has been shown to elicit a stabilizing effect to the double stranded DNA structure (Rodríguez López et al., 2010a); a feature that accords with its reported effect on the fine structure of DNA (Heinemann and Hahn, 1992). In contrast, spectroscopic (Thalhammer et al., 2011), calorimetric (Wanunu et al., 2011), and HRM (Rodríguez López et al., 2012a) analyses have all suggested that presence of the alternate base modifications (5hmC and m6A) in the DNA could reverse the stabilizing effect of 5mC. Whether or not the changes to DNA thermostability induced by 5hmC have functional impact on gene expression is still a matter of conjecture. Certainly, some authors have reported that 5mC hydroxylation is associated with the activation of gene transcription (Ito et al., 2010; Thalhammer et al., 2011; Wanunu et al., 2011) while others argue that any contribution to transcriptional activation or repression is highly context-dependent (Wu et al., 2011). Whatever role (if any) that these alternate base modifications play in gene regulation, it is already clear that they are far less abundant, if present at all, in the plant genome than 5mC and so probably hold only limited value as diagnostic marks for epifingerprinting purposes. It is therefore the distribution of 5mC in the genome that has formed the focus of attempts to link epifingerprints to the physiological, developmental, or stress status of higher organisms, including crop plants.

An array of methods has been developed to describe the global pattern of 5mC across the genome (for extensive reviews on the subject, see Tost and Gut, 2009; Chaudhry, 2010; Plongthongkum et al., 2014). All methods carry their own limitations (Rodríguez López et al., 2010a) but can be broadly grouped into three functional types that: (1) indicate the methylation status of a specific sequence; (2) reveal the degree and patterning of DNA methylation across partly characterized genomes; (3) facilitate the discovery and sequencing of new epialleles (Fraga and Esteller, 2002; Dahl and Guldberg, 2003).

## The Potential Value of Epi-Fingerprinting for Agriculture

### Epi-Fingerprinting of *In Vitro* Cultured Plant Material

The ability to propagate elite or desirable clones is an essential part of the seed production industry. The advent of reliable *in vitro* systems for the replication and regeneration of plant materials has led to their widespread deployment for propagation (Bertrand et al., 2011; Etienne et al., 2012), germplasm conservation (Fang et al., 2009), and breeding purposes (Henry, 1998), as well as for more fundamental research on model species (Berdasco et al., 2008; De-la-Peña et al., 2012; Moricová et al., 2013). For micropropagation and genetic transformation systems to be efficient, it is necessary that the plants recovered from them are genetically and epigenetically faithful to the original stock material. Trueness-to-type is of particular importance when propagating elite genotypes of high value crops such as grapevine: especially in traditional vine areas where high clone quality is a prerequisite (Schellenbaum et al., 2008). In comparison to genetic somaclonal variation, divergence between DNA methylation patterns is generally wider among regenerated plants and can be directly associated with ‘plastic’ phenotypic variation (Miguel and Marum, 2011). The loss of epigenetic fidelity during micropropagation has been a major source of economic damage in several crops. For instance, in oil palm, mantled inflorescence syndrome was found to be associated with global changes to *C*-methylation status during micropropagation, and caused catastrophic reductions in yield among all affected plants and incurred huge costs to the industry (Matthes et al., 2001). Many studies have reported global changes to the distribution of cytosine methylation can be induced by *in vitro* culture spanning an impressive array of species in a wide taxonomic spread. Examples include: tobacco (*Nicotiana tabacum*, Schmitt et al., 1997); rice (*Oryza sativa*, Xiong et al., 1999); strawberry (*Fragaria Xananassa*, Hao et al., 2002); potato (*Solanum tuberosum*, Joyce and Cassells, 2002; *A. thaliana*, Bardini et al., 2003); oil palm (*Elaeis guineensis*, Jaligot et al., 2004); and cocoa (*Theobroma cacao*, Rodríguez López et al., 2010b). On the other hand, some forms of such ‘somaclonal variation’ may offer a source of valuable new variation that has potential applications in plant breeding (Henry, 1998). Furthermore, different studies have shown that epigenetic regulation plays an important role during plant development *in vitro* (Rodríguez López et al., 2010b; Nic-Can et al., 2013). Regardless of whether the change in methylation status evokes a desirable or unwanted outcome, there is clearly great value in the ability to detect these changes or at least to predict the scale of any phenotypic or physiological divergence. Advances in our understanding of the links between gene expression and phenotype mean that the ambition may now turn from simply viewing these plants as a new source of variation for breeding and toward a more targeted approach that deliberately manipulates the process for use in crop improvement efforts.

### Epi-Fingerprinting for Breeding and Varietal Selection

The majority of agricultural land is cultivated with commodity crops that are either highly inbred or clonal. These genetically invariant populations nevertheless exhibit measurable morphological or developmental plasticity, even when grown under controlled conditions, which may be at least partly explained by stable epigenomic states (Hauben et al., 2009). It has recently been argued that these epigenetic sources of variation may even be greater than those attributable to genetic causes (Hirsch et al., 2013; Schmitz et al., 2013). Several authors have linked genotype-specific changes to DNA methylation to yield components or to other agronomically desirable traits (e.g., Gourcilleau et al., 2010; Alonso et al., 2014; **Table 1**). The first classic example of a single epiallelic gene variant was attributed to hypermethylation of the CYCLOIDEA gene of *Linaria vulgaris*; a state which causes radial symmetry of previously bilaterally symmetric flowers (Cubas et al., 1999). Other epigenetic variants have subsequently emerged with features that have economic potential. For instance, the hypomethylation of the rice gene FIE1 induces its ectopic expression and results in a dwarf and flower-aberrant phenotype (Zhang et al., 2012). Goettel and Messing (2013) reported that cytosine methylation of a gene (P1-rr) encoding for a Myb-like transcription factor that mediates pigmentation in floral organs and grains, is negatively correlated with transcription and pigment levels. These mutations are thought to have arisen spontaneously by somatic epi-mutation and later became fixed after repeated passage through meiosis.

**Table 1:** Examples of plant genes involved in agronomic traits affected by DNA methylation.

Epiallele type	Locus	Epigenetic regulation	Trait	Reference
	LRR	Regulated by DNA methylation	Disease resistance	Yu et al. (2013)
	Plastocyanin-like domain	Differentially methylated	Low pH and aluminium stress in sorghum	Kimatu et al. (2011)
	CaLB domain family protein	Possible regulation by DNA methylation	Abiotic stress signaling	Dubin et al. (2014)
	BALL (BAL)		Pathogen resistance	Stokes et al. (2002)
	CIPK		Abiotic stress response in plants	Shan et al. (2013)
Stress response epialles	CBS domain-containing protein Phosphoribulokinase/Uridine kinase family	Differentially methylated under cold stress	Abiotic stress response in plants Photosynthesis and energy metabolism	Shan et al. (2013) Pan et al. (2011)
	SPEECHLESS	Differentially methylated under low relative humidity	Stomata development control	Tricker et al. (2012)
	FAMA	Differentially methylated under low relative humidity	Stomata development control	Tricker et al. (2012)
	NtGPDL	Differentially methylated under abiotic stress	Abiotic stress response in plants	Choi and Sano (2007)
	CRK8	Methylated in rice/Differentially methylated in maize under cold stress	Transposon	Shan et al. (2013)
	CWF19	Methylated	Cell cycle control protein	Jeon et al. (2015)
	EMB71 (MAPKKK4, YDA)	Possible regulation by DNA methylation	Embryo and in stomata developmental programmes	Tricker personal communication
Developmentally	ARF2	Target for sRNA	Repressor of cell division and organ growth	Zhang et al. (2014)
regulated epialleles	GT-2 related proteins	Regulated by DNA methylation	Organ morphogenesis	Song et al. (2012)
	MEG1 Cys-rich protein	Maternal parent-of-origin expression	Regulates seed development in maize	Gutierrez-Marcos et al. (2004)
	MEA Polycomb protein		Regulates seed development in maize	Grossniklaus et al. (1998)
	FIS2 Transcription factor		Regulates seed development in maize	Luo et al. (1999)
	FIE Polycomb protein		Regulates seed development in maize	Ohad et al. (1996)
	PHERES1 MADS TF		Regulates seed development in maize	Kohler and Makarevich (2006)
	MPC Poly(A) binding protein			Tiwari et al. (2008)
	FWA	Maternally imprinted	Positive regulator of flowering	Kinoshita et al. (2004)
	SUPERMAN	Hypermethylation induce mutant floral morphologies	Regualtion of floral whorls development	Jacobsen and Meyerowitz (1997)
	AGAMOUS	Hypermethylation induce mutant floral morphologies	Regualtion of floral whorls development	Ito (2012)
	RIN	Differentially methylated during fruit development	Regulator of shelf life and quality	Zhong et al. (2013)
	NOR	Differentially methylated during fruit development	Regulator of shelf life and quality	Zhong et al. (2013)
	PG2A	Differentially methylated during fruit development	Regulator of shelf life and quality	Zhong et al. (2013)
	PSY	Differentially methylated during fruit development	Regulator fruit color	Zhong et al. (2013)
	PDS	Differentially methylated during fruit development	Regulator fruit color	Zhong et al. (2013)
			
	pectinesterase-1	Differentially methylated under cold stress in maize	ABA signalling	Shan et al. (2013)
	Hexokinase	Differentially methylated under cold stress	Meiosis	Shan et al. (2013)
	MADS-box protein	Differentially methylated under cold stress in maize	Cell wall formation	Shan et al. (2013)
	Kinesin-like protein	Differentially methylated under cold stress in maize	Flowering time	Shan et al. (2013)
	*NAM*	Hypomethylation-dependent up-regulation in pluripotent protoplasts	Determining the pluripotent state of the cells	Avivi et al. (2004)
	ATAF1	Hypomethylation-dependent up-regulation in pluripotent protoplasts	Determining the pluripotent state of the cells	Avivi et al. (2004)
	CUC2	Hypomethylation-dependent up-regulation in pluripotent protoplasts	Determining the pluripotent state of the cells	Avivi et al. (2004)
	*MAPK12*	Hypermethylated in callus and cell suspensions		Berdasco et al. (2008)
*In vitro* culture induced epialleles	GSTU10	Hypermethylated in callus and cell suspensions		Berdasco et al. (2008)
	BXL1	Hypermethylated in callus and cell suspensions		Berdasco et al. (2008)
	TTG1	Hypermethylated in cell suspensions		Berdasco et al. (2008)
	GSTF5	Hypermethylated in cell suspensions		Berdasco et al. (2008)
	SUVH8	Hypermethylated in cell suspensions	Regulates rippening	Berdasco et al. (2008)
	CNR	Spontaneous epiallele in tomato		Manning et al. (2006)
	SP11	Regulated by Methylation	Sporophytic self-incompatibility system	Shiba et al. (2006)
Natural epialleles	CYCLOIDEA	Hypermethylated mutant	Control floral symetry *Linaria vulgaris*	Cubas et al. (1999)
	P1	Hypermethylated alleles P-pr-1 and P-pr-2	Grain colour in maize	Das and Messing (1994)
	D1	Hypermethylated in metastable Epi-d1	Plant height in rice	Miura et al. (2009)
	GUN4	Regulated by DNA methylation in rice	Chlorophyll biosynthesis	Li et al. (2014)
	XTH1	Regulated by DNA methylation in potato		Da et al. (2012)

Systematic selection for fixed epi-loci is not the only possible source of new varietal material with potential to improve crop production or quality. Environmentally induced epi-alleles also offer an important potential source of exploitable variation. For many inbreeding and clonal crops, environmentally induced epigenetic variation can sometimes outweigh genetic variation, with such changes being induced by exposure to various aspects of the living environment (Raj et al., 2011; Tricker et al., 2012; Hirsch et al., 2013). These properties can lead to an epigenetic convergence of populations when grown under similar conditions (Schulz et al., 2014) but can also lead to spontaneous divergence of fixed epigenetic states (Becker et al., 2011). Tricker et al. (2013b) proposed an approach in which the deliberate manipulation of the specific aspects of the growing environment could be used to induce desirable changes in tolerance to low humidity and periodic drought. Nevertheless, the disentanglement of this kind of epigenetic variation from the genetic background that underpins the capacity to produce new variability continues to pose major technical difficulties (Cortijo et al., 2014) and is probably still some way from commercial reality.

For vegetatively propagated perennial crops such as grapevine (Zufferey et al., 2000) or *Pinus radiata* (Fraga et al., 2001) the need to fix between generations is circumvented. For these crops there is a long association between productivity and quality characteristics and plant age. The possibility that this relationship has an epigenetic basis and so is amiable for manipulation is especially appealing. Certainly, it is known that DNA methylation changes progressively during maturation and aging, for both plants and animals species (Theiss and Follmann, 1980; Quemada et al., 1987; Fraga et al., 2005). There is also evidence that these changes are associated with altered expression of genes that are implicated in morphological changes in plants (Galaud et al., 1993) and animals (Zhang et al., 2002). More specifically, the extent of genomic DNA methylation in pine is a strong indicator of aging and can provide molecular evidence of reinvigoration (Fraga et al., 2001). Thus, there is scope to manipulate the methylation status of crop genomes either chemically using methyltransferase inhibitors, by exposure to signaling molecules or by manipulation of the growing environment. Individuals exhibiting stable, rejuvenated methylation profiles, and associated phenotypes could then be selected and used for commercial planting.

### Epi-Fingerprinting as an Indicator of Plant Health

In addition to the generation of new variation there is also considerable scope for deploying epigenetic fingerprinting approaches to improve the efficacy of agronomic or prophylactic interventions. Plants are sessile organisms and so unable to avoid abiotic or biotic stresses. They must instead rely on rapid and effective stress response systems to withstand harmful changes to the living environment to enhance their chances of survival. Plants have amassed an array of mechanisms for detecting and then responding to stresses in ways that can include substantial amendments to key metabolic pathways (Madlung and Comai, 2004). Such responses can be activated in a number of ways including the adjustment of the transcriptional control of genes through differential cytosine methylation (Aceituno et al., 2008).

Several authors have noted that large numbers of biotic and abiotic stresses induce global changes to the methylation patterns of plants (Stokes et al., 2002; Boyko and Kovalchuk, 2008, 2011; Chinnusamy and Zhu, 2009). This feature means there is often a clear relationship between the detection of a particular stress by a plant and overall *C*-methylation profile. This property means that there is scope for the use of *C*-methylation fingerprinting approaches as a tool to diagnose the early onset or asymptomatic exposure of a crop to a range of stresses. Several workers have demonstrated that diagnostic changes in methylation fingerprints are associated with exposure to a wide range of abiotic stresses including drought (Raj et al., 2011; Tricker et al., 2013b), low relative humidity, (Tricker et al., 2012), low temperatures (Pan et al., 2011), salt and heavy metals (Choi and Sano, 2007; Verhoeven et al., 2010), and low nutrient levels (Verhoeven et al., 2010). The same is seemingly also true for exposure to biotic stresses, with changed DNA methylation profiles also being reported following plant–herbivore (Verhoeven et al., 2010, Herrera and Bazaga, 2013) and plant–pathogen interactions (Mason et al., 2008; Boyko and Kovalchuk, 2011). These observations have yet to be used as a basis to develop a robust set of methylation markers to routinely diagnose exposure of crops to these stresses but this aspiration appears both attractive and tractable within a relatively short time period.

There is also opportunity to use *C*-methylation profiling to gain better understanding of the relationship between the stress and the physiological response of the plant to that stress. Herrera and Bazaga (2013) reported that phenotypic changes adopted by the plant in response to stress (such as prickly leaves induced by herbivory) positively correlated to global changes in DNA methylation. Resistance to *Rhizoctonia solani* in maize is similarly linked to global shifts in DNA methylation (Li et al., 2011). Sequence characterisation of these differentially methylated loci may ultimately provide a useful route through which to discover candidate genes that are implicated in these responses. This approach has been adopted in other cases. For instance in rice, where resistance to bacterial blight is linked to plant age, it has been shown that acquired resistance is regulated by the hypo/hypermethylation of several loci. Such methylation changes correlate with the expression levels of several genes including a putative Gag-Pol polyprotein, a putative RNA helicase of the Ski2 subfamily and a putative receptor-like protein kinase (Sha et al., 2005). There has also been interest in tracking changes in DNA methylation associated with virus silencing in plants (English et al., 1996).

The apparent stability of some *C*-methylation sites following induction allows for stress detection long after initial exposure and means that carefully selected epimarkers potentially provide a more robust source of *a posteriori* stress diagnosis than more ephemeral changes within the cell such as the abundance of mRNA (transcriptomics), proteins (proteomics), or metabolites (metabolomics). Furthermore, this ‘memory of stress’ is not limited to cells and cell lineages but as described above can also persist through filial generations. Boyko and Kovalchuk (2011) showed that changes to the DNA methylation patterns of plants associated with continuous interactions with pathogens were successfully transmitted and fixed in their progeny seemingly also potentially allowing for the diagnosis of parental stress exposure.

Looking ahead, it seems inevitable that in the relatively near future there will be methylation markers developed for many crops able to track developmental progression and also the exposure and response of the plants to the stresses they are experiencing. The long-term possibility of using these markers as sentinels of health and developmental state leads to the enticing prospect that they may ultimately be integrated into models to predict yield. If applied onto a broader scale, it is even possible that epigenetic fingerprinting of airborne pollen samples for signatures of stress could eventually augment existing monitoring of the landscape for the effects of climate change or to track new epidemiological events, and so facilitate more timely and targeted interventions.

### Epi-Fingerprinting and Product Quality

The high market value of ‘top end’ agricultural products used for nutritional or medicinal properties frequently attracts fraudulent labeling of lesser products with lower quality or commercial value (Mader et al., 2011). Certifying the authenticity and origin of such products is a legal requirement in many jurisdictions to avoid unfair competition and assure consumers protection against fraudulent practices (Reid et al., 2006). Although there is an increasing demand by consumers for high quality food products (Luykx and van Ruth, 2008), the majority of authentication techniques for food products have focussed on species or varietal identification or on the chemical composition of processed foods (Sentandreu and Sentandreu, 2011). However, quality traits of plant products are not only influenced by the choice of species or cultivars. In some agricultural products, quality can be primarily determined by the harvested components of the crop used to generate a product (Srancikova et al., 2013) or else by climate, location, crop age, management systems used to cultivate the crop (e.g., industrialized versus organic farming, manure versus *N*-fertilizer; Posner et al., 2008). Equally, soil conditions, as well as the interactions of different environmental conditions or “terroir” can be viewed as important quality determinants of products such as wine (van Leeuwen et al., 2004). These conditions affect plant composition variables such as dry matter content and furthermore starch, crude protein, amino acids, nitrate, sugars, and citric acid (Müller and Hippe, 1987). The measurement of such components has often necessitated development of a series of independent tests to detect fraudulent labeling. The use of methylation profiles as a diagnostic tool relating to several different aspects of crop quality is therefore appealing because it provides a ‘plant’s perspective of the growing environment.’ This area of methylation profiling is still untested but would be especially alluring if evidence can be provided to distinguish between agronomic practices (such as those used for organic farming) that are currently primarily verified only by certification.

New evidence is now emerging to suggest that this may be possible. For example, Boyko et al. (2010) showed that exposure of *A. thaliana* to a range of mild abiotic modifications (salt around the roots, UVC, cold, heat, and flood) could be detected by reproducible changes in DNA methylation patterns. Similarly, in clonally propagated poplar grown under different conditions of water availability, differences in genome-wide DNA methylation paralleled differences in transcriptome, suggesting an epigenomic basis for the clone history-dependent divergence (Raj et al., 2011) and illustrating the plausibility of epigenetic profiling to characterize watering regime. Indeed, cultivation conditions of a wide variety of plants have now been shown to induce differences at methylome level (i.e., Dandelions, Verhoeven et al., 2010; mangrove, Lira-Medeiros et al., 2010; alligator weed, Li et al., 2013). These findings open the door for deploying epigenetic profiling approaches to diagnose growth conditions and geographical region of origin of otherwise identical crops and theirs processed products.

It is therefore tempting to speculate that quality traits associated with crop management may also be detectable using the same *C*-methylation markers. There is equally scope also to differentiate between products generated from parts of the plant with different market value. Certainly, it is now well established that different cell types or tissues within an organism can have markedly different methylation profiles (Baron et al., 2006; Feng et al., 2010; Rodríguez López et al., 2010b, 2012b) and that the use of epigenetic markers has been proven to be an effective means of generating organ-specific epigenetic markers as a tool for identifying the tissue of origin in plant (Rodríguez López et al., 2010b) and animal (Rodríguez López et al., 2012b) products. This gives rise to the prospect of simplifying global methylation patterns to generate generate smaller numbers of highly diagnostic epimarkers for use in food quality assessment. Such markers could not only have potential value in identifying the cultivating system and product composition, but also to other factors affecting quality such as storage, transport and processing conditions.

## Conclusion

Epigenetic control mechanisms provide the crop plant with an ability to respond to the many and varied challenges posed to them by an ever-changing growing environment during growth and development. Of all these mechanisms, histone tail modifications and DNA methylation are by far the better studied. Of the two, DNA methylation way of action is the better understood, the easier to analyze and the one with most associated epialleles in the literature.

We have shown that the deliberate manipulation of this relationship through direct (chemical) and indirect (environmental) interventions holds the potential to generate new and useful variability to the crop. In some cases the induced changes can alter the genome regulatory system of the crop in such a way as to allow it to better cope with particular, anticipated stress types. The capability to fix at least some of these states across generations offers the tantalizing possibility of a targeted system of epigenetic breeding to augment existing breeding efforts, and has particular appeal for long-lived clonal crops. We have also shown that gaining a better understanding of the relationship between the stress elicitor and the changed epigenetic state offers new opportunities for the identification of candidate genes that are important in conferring resilience against important stresses. Such stable epigenetic markers, especially if associated to commercially interesting traits, can be of interest to plant breeders. Apart from variations in the gene sequence, epigenetic variation may contribute to commercially interesting traits.

**FIGURE 2:**
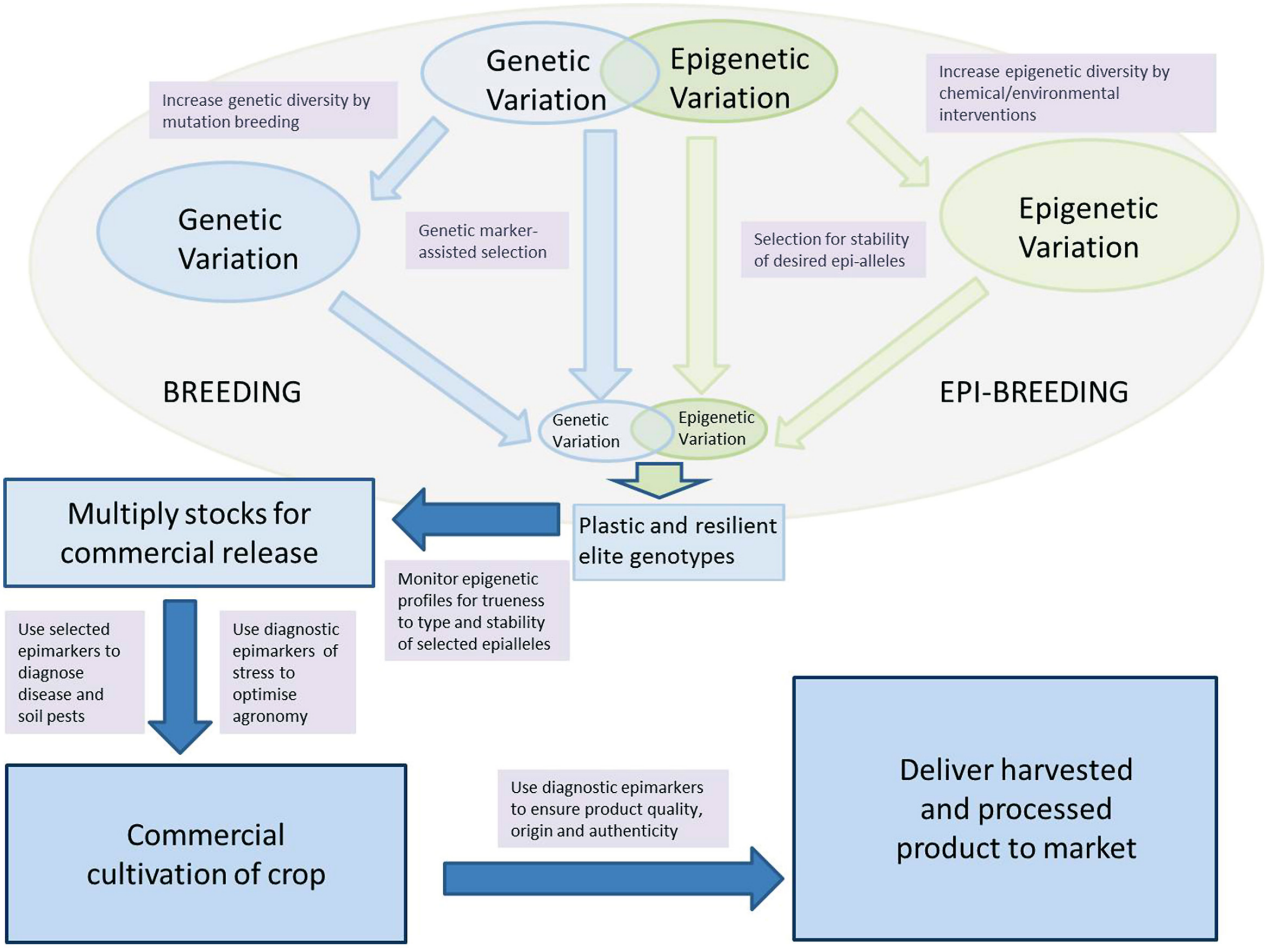
**Epi-fingerprinting and epi-interventions for improved crop production and food quality: schematic illustration on how epi-fingerprinting and epigenetic interventions could potentially impact on various parts of the agricultural supply chain**. The model starts with a combined breeding and epi-breeding approach to varietal production and is followed by seed/clone multiplication systems that uses epigenetic profiling techniques to minimize appearance of off-types. Cultivation of the crop is augmented by agronomic and pest/disease management strategies that utilize epi-fingerprinting to diagnose/optimize the health status of the crop. At delivery to market, epi-fingerprinting is used to authenticate products and to ensure quality.

However, it is perhaps as a diagnostic tool of stress that there is the greatest source of unexplored opportunity for short-term step improvements to crop management and production. A plethora of works have shown that there is a clear and strong relationship between a vast array of stresses and the *C*-methylation status of crop plants. Conversion of these global differences into specific diagnostic epimarks of stress detection and stress-induced physiological response by the crop plants offers a range of opportunities for the improvement in varietal selection, crop management, for the control of pests and disease, and to control and regulate the quality of agricultural products. Moreover, the methylome epifingerprinting can be considered as a measure of the phenotype of the crop’s genome. Such an ‘epiphenotype’ not only provides a new diagnostic tool to study stress responses and developmental progression but also provides a useful bridge that allows direct functional relationships to be inferred between the growing environment and associated genome regulation. In the medium term we expect the collective impact of these developments to enable substantive advances in crop production and protection; an epigreen revolution (**Figure 2**).

## Conflict of Interest Statement

The authors declare that the research was conducted in the absence of any commercial or financial relationships that could be construed as a potential conflict of interest.

## References

[B1] Aceituno F. F. Nick M. Seung Y. R. Rodrigo A. G. (2008). The rules of gene expression in plants: organ identity and gene body methylation are key factors for regulation of gene expression in *Arabidopsis thaliana*. *BMC Genomics* 9:438–451. 10.1186/1471-2164-9-438 18811951 PMC2566314

[B2] Agorio A. Vera P. (2007). ARGONAUTE4 is required for resistance to *Pseudomonas syringae* in *Arabidopsis*. *Plant Cell* 198 3778–3790. 10.1105/tpc.107.054494 17993621 PMC2174867

[B3] Alonso C. Perez R. Bazaga P. Medrano M. Herrera C. M. (2014). Individual variation in size and fecundity is correlated with differences in global DNA cytosine methylation in the perennial herb *Helleborusfoetidus* (Ranunculaceae). *Am. J. Bot.* 101 1309–1313. 10.3732/ajb.1400126 25143467

[B4] Amoah S. Kurup S. Rodríguez López C. M. Welham S. J. Powers S. J. Hopkins C. J. (2012). A hypomethylated population of *Brassica rapa* for Forward and reverse Epi-Genetics. *BMC Plant Biol.* 12:193–210. 10.1186/1471-2229-12-193 23082790 PMC3507869

[B5] Ashapkin V. V. Kutueva L. I. Vanyushin B. F. (2002). The gene for domains rearranged methyltransferase (DRM2) in *Arabidopsis thaliana* plants is methylated at both cytosine and adenine residues. *FEBS Lett.* 532 367–372. 10.1016/S0014-5793(02)03711-0 12482594

[B6] Avivi Y. Morad V. Ben-Meir H. Zhao J. Kashkush K. Tzfira T. (2004). Reorganization of specific chromosomal domains and activation of silent genes in plant cells acquiring pluripotentiality. *Dev. Dyn.* 230 12–22. 10.1002/dvdy.20006 15108305

[B7] Ball P. (2013). DNA: celebrate the unknowns. *Nature* 496 419–420. 10.1038/496419a 23619675

[B8] Bardini M. Labra M. Winfield M. Sala F. (2003). Antibiotic-induced DNA methylation changes in calluses of *Arabidopsis thaliana*. *Plant Cell Tissue Organ Cult.* 72 157–162. 10.1023/A:1022208302819

[B9] Baron U. Türbachova I. Hellwag A. Eckhardt F. Berlin K. Hoffmuller U. (2006). DNA methylation analysis as a tool for cell typing. *Epigenetics* 1 55–60. 10.4161/epi.1.1.2643 17998806

[B10] Becker C. Hagmann J. Muller J. Koenig D. Stegle O. Borgwardt K. (2011). Spontaneous epigenetic variation in the *Arabidopsis thaliana* methylome. *Nature* 480 245–249. 10.1038/nature10555 22057020

[B11] Becker C. Weigel D. (2012). Epigenetic variation: origin and transgenerational inheritance. *Curr. Opin. Plant Biol* 15 562–567. 10.1016/j.pbi.2012.08.004 22939250

[B12] Berdasco M. Alcazar R. García-Ortiz M. V. Ballestar E. Fernández A. F. Teresa Roldán-Arjona T. (2008). Promoter DNA hypermethylation and gene repression in undifferentiated *Arabidopsis* cells. *PLoS ONE* 3:e3306 10.1371/journal.pone0003306.g003 PMC2556100 18827894

[B13] Berger S. L. (2007). The complex language of chromatin regulation during transcription. *Nature* 447 407–412. 10.1038/nature05915 17522673

[B14] Bertrand B. Alpizar E. Llara L. Santacreo R. Hidalgo M. Quijano J. M. (2011). Performance of *Coffea arabica* F1 hybrids in agroforestry and full-sun cropping systems in comparison with pure lines varieties. *Euphytica* 181 147–158. 10.1007/s10681-011-0372-7

[B15] Bird A. (2007). Perceptions of epigenetics. *Nature* 447 396–398. 10.1038/nature05913 17522671

[B16] Boyko A. Blevins T. Yao Y. Golubov A. Bilichak A. Ilnytskyy Y. (2010). Transgenerational adaptation of *Arabidopsis* to stress requires DNA methylation and the function of Dicer-like proteins. *PLoS ONE* 5:e9514 10.1371/journal.pone.0009514 PMC2831073 20209086

[B17] Boyko A. Kathiria P. Zemp F. J. Yao Y. Pogribny I. Kovalchuk I. (2007). Transgenerational changes in the genome stability and methylation in pathogen-infected plants: (virus-induced plant genome instability). *Nucleic Acids Res.* 35 1714–1725. 10.1093/nar/gkm029 17311811 PMC1865051

[B18] Boyko A. Kovalchuk I. (2008). Epigenetic control of plant stress response. *Environ. Mol. Mutagen.* 49 61–72. 10.1002/em.20347 17948278

[B19] Boyko A. Kovalchuk I. (2011). Genetic and epigenetic effects of plant–pathogen interactions: an evolutionary perspective. *Mol. Plant* 4 1014–1023. 10.1093/mp/ssr022 21459830

[B20] Bräutigam K. Vining K. J. Lafon-Placette C. Fossdal C. G. Mirouze M. Gutiérrez Marcos J. (2013). Epigenetic regulation of adaptive responses of forest tree species to the environment. *Ecol. Evol.* 3 399–415. 10.1002/ece3.461 23467802 PMC3586649

[B21] Breto M. P. Ruiz C. Pina J. A. Asíns M. J. (2001). The diversification of citrus clementina hort. ex tan., a vegetatively propagated crop species. *Mol. Phylogen. Evol.* 21 285–293. 10.1006/mpev.2001.1008 11697922

[B22] Chahwan R. Wontakal S. N. Roa S. (2011). The multidimensional nature of epigenetic information and its role in disease. *Discov. Med.* 11 233–243. 21447282

[B23] Chaudhry M. A. (2010). Strategies for detecting genomic DNA methylation: a survey of US patents. *Recent Pat. DNA Gene. Seq*. 4 79–85. 10.2174/187221510793205719 20426762

[B24] Chinnusamy V. Zhu J.-K. (2009). Epigenetic regulation of stress responses in plants. *Curr. Opin. Plant Biol.* 12 133–139. 10.1016/j.pbi.2008.12.006 19179104 PMC3139470

[B25] Choi C.-S. Sano H. (2007). Abiotic-stress induces demethylation and transcriptional activation of a gene encoding a glycerophosphodiesterase-like protein in tobacco plants. *Mol. Genet. Genomics* 277 589–600. 10.1007/s00438-007-0209-1 17273870

[B26] Collard B. C. Y. Mackill D. J. (2008). Marker-assisted selection: an approach for precision plant breeding in the twenty-first century. *Philos. Trans. R. Soc. Lond. B Biol. Sci.* 363 557–572. 10.1098/rstb.2007.2170 17715053 PMC2610170

[B27] Conrath U. (2011). Molecular aspects of defence priming. *Trends Plant Sci.* 16 524–531. 10.1016/j.tplants.2011.06.004 21782492

[B28] Cortijo S. Wardenaar R. Colomé-Tatché M. Gilly A. Etcheverry M. Labadie K. (2014). Mapping the epigenetic basis of complex traits. *Science* 343 1145–1148. 10.1126/science.1248127 24505129

[B29] Cubas P. Vincent C. Coen E. (1999). An epigenetic mutation responsible for natural variation in floral symmetry. *Nature* 401 157–161. 10.1038/43657 10490023

[B30] Da K. Nowak J. Flinn B. (2012). Potato cytosine methylation and gene expression changes induced by a beneficial bacterial endophyte, *Burkholderia phytofirmans* strain PsJN. *Plant Physiol. Biochem.* 50:24e34 10.1016/j.plaphy.2011.09.013 22099516

[B31] Dahl C. Guldberg P. (2003). DNA methylation analysis techniques. *Biogerontology* 4 233–250. 10.1023/A:1025103319328 14501188

[B32] Das O. P. Messing J. (1994). Variegated phenotype and developmental methylation changes of a maize allele originating from epimutation. *Genetics* 136 1121–1141. 8005419 10.1093/genetics/136.3.1121 PMC1205868

[B33] Daymond A. J. Tricker P. J. Hadley P. (2011). Genotypic variation in photosynthesis in cacao is correlated with stomatal conductance and leaf nitrogen. *Biol. Plant.* 55 99–104. 10.1007/s10535-011-0013-y

[B34] De-la-Peña C. Nic-Can G. I. Ojeda G. Herrera-Herrera J. L. López-Torres A. Wrobel K. (2012). KNOX1 is expressed and epigenetically regulated during in vitro conditions in *Agave* spp. *BMC Plant Biol*. 12:203–214. 10.1186/1471-2229-12-203 23126409 PMC3541254

[B35] Dubin M. J. Zhang P. Meng D. Remigereau M.-S. Osborne E. J. Casale F. P. (2014). DNA methylation variation in *Arabidopsis* has a genetic basis and shows evidence of local adaptation. *Elife* 4:e05255 10.7554/eLife.05255 PMC4413256 25939354

[B36] Dunn D. B. Smith J. D. (1955). Occurrence of a new base in the deoxyribonucleic acid of a strain of *Bacterium Coli*. *Nature* 175 336–337. 10.1038/175336a0 13235889

[B37] English J. J. Mueller E. Baulcombe D. C. (1996). Suppression of virus accumulation in transgenic plants exhibiting silencing of nuclear genes. *Plant Cell* 8 179–188. 10.1105/tpc.8.2.179 12239381 PMC161090

[B38] Erdmann R. M. Souza A. L. Clish C. B. Gehring M. (2015). 5-Hydroxymethylcytosine is not present in appreciable quantities in *Arabidopsis* DNA. *G3* (*Bethesda*) 5 1–8. 10.1534/g3.114.014670 25380728 PMC4291460

[B39] Etienne H. Bertrand B. Montagnon C. Bobadilla Landey R. Dechamp E. Jourdan I. (2012). Un exemple de transfert technologique réussi en micropropagation: la multiplication de *Coffea arabica* parembryogenèse somatique. *Cah. Agric.* 21 115–124.

[B40] Evenson R. E. Gollin D. (2000). Assessing the impact of the green revolution, 1960 to 2000. *Science* 300 758–762. 10.1126/science.1078710 12730592

[B41] Fang J.-Y. Wetten A. Adu-Gyamfi R. Wilkinson M. Rodríguez-López C. (2009). Use of secondary somatic embryos promotes genetic fidelity in cryopreservation of cocoa (*Theobroma cacao* L.). *Agric. Food Sci.* 18 152–159. 10.2137/145960609789267579

[B42] Feng S. Jacobsen S. E. Reik W. (2010). Epigenetic reprogramming in plant and animal development. *Science* 330 622–627. 10.1126/science.1190614 21030646 PMC2989926

[B43] Fraga M. F. Ballestar E. Paz M. F. Ropero S. Setien F. Ballestar M. L. (2005). Epigenetic differences arise during the lifetime of monozygotic twins. *Proc. Natl. Acad. Sci. U.S.A.* 102 10604–10609. 10.1073/pnas.0500398102 16009939 PMC1174919

[B44] Fraga M. F. Esteller M. (2002). DNA Methylation: a profile of methods and applications. *BioTechniques* 33 632–649. 10.3389/fgene.2011.00074 12238773

[B45] Fraga M. F. Rodriguez R. Canal M. J. (2001). Genomic DNA methylation–demethylation during aging and reinvigoration of *Pinus radiata*. *Tree Physiol.* 22 813–816. 10.1093/treephys/22.11.813 12184986

[B46] Galaud J.-P. Gaspar T. Boyer N. (1993). Effect of anti-DNA methylation drugs on growth, level of methylated DNA, peroxidase activity and ethylene production of *Bryonia dioica* internodes. *Physiol. Plant.* 87 528–534. 10.1111/j.1399-3054.1993.tb02503.x

[B47] Geyer K. K. Rodríguez López C. M. Heald J. Wilkinson M. J. Hoffmann K. F. (2011). Cytosine methylation regulates oviposition in the pathogenic blood fluke *Schistosoma mansoni*. *Nat. Commun.* 9 424–434. 10.1038/ncomms1433 21829186 PMC3265374

[B48] Goettel W. Messing J. (2013). Paramutagenicity of a p1 epiallele in maize. *Theor Appl Genet.* 126 159–177. 10.1007/s00122-012-1970-z 22986680

[B49] Gourcilleau D. Bogeat-Triboulot M. B. Le Thiec D. Lafon-Placette C. Delaunay A. El-Soud W. A. (2010). DNA methylation and histone acetylation: genotypic variations in hybrid poplars, impact of water deficit and relationships with productivity. *Ann. For. Sci.* 67 208–217. 10.1051/forest/2009101

[B50] Grant-Downton R. T. Dickinson H. G. (2005). Epigenetics and its implications for plant biology: 1. The epigenetic network in plants. *Ann. Bot.* 96 1143–1164. 10.1093/aob/mci273 16254022 PMC4247072

[B51] Grossniklaus U. Vielle-Calzada J. P. Hoeppner M. A. Gagliano W. B. (1998). Maternal control of embryogenesis by medea, a Polycomb group gene in *Arabidopsis*. *Science* 280 446–450. 10.1126/science.280.5362.446 9545225

[B52] Gutierrez-Marcos J. F. Costa L. M. Biderre-Petit C. Khbaya B. O’Sullivan D. M. Wormald M. (2004). Maternally expressed gene 1 is a novel maize endosperm transfer cell-specific gene with a maternal parent-of-origin pattern of expression. *Plant Cell* 16 1288–1301. 10.1105/tpc.019778 15105441 PMC423216

[B53] Hao Y. J. You C. X. Deng X. X. (2002). Analysis of ploidy and the patterns of amplified length polymorphism and methylation sensitive amplification polymorphism in strawberry plants recovered from cryopreservation. *Cryoletters* 23 37–46. 11912506

[B54] Hattman S. (2005). DNA-[Adenine] methylation in lower eukaryotes. *Biochemistry* (*Moscow*) 70 550–558. 10.1007/s10541-005-0148-6 15948708

[B55] Hauben M. Haesendonckx B. Standaert E. Van Der Kelen K. Azmi A. Akpo H. (2009). Energy use efficiency is characterized by an epigenetic component that can be directed through artificial selection to increase yield. *Proc. Natl. Acad. Sci. U.S.A.* 106 20109–20114. 10.1073/pnas.0908755106 19897729 PMC2774259

[B56] Heil M. Ton J. (2008). Long-distance signalling in plant defence. *Trends Plant Sci.* 13 264–272. 10.1016/j.tplants.2008.03.005 18487073

[B57] Heinemann U. Hahn M. (1992). C-C-A-G-G-C-m5C-T-G-G. Helical fine structure, hydration, and comparison with C-C-A-G-G-C-C-T-G-G. *J. Biol. Chem.* 267 7332–7341. 1559976

[B58] Henderson I. R. Jacobsen S. E. (2007). Epigenetic inheritance in plants. *Nature* 447 418–424. 10.1038/nature05917 17522675

[B59] Henry R. J. (1998). “Molecular and biochemical characterization of somaclonal variation,” in *Somaclonal Variation and Induced Mutations in Crop Improvement*, eds Jain S. M. Brar D. S. Ahloowalia B. S. (Dordrecht: Kluwer Academic Publisher), 485–499. 10.1007/978-94-015-9125-6_24

[B60] Herrera C. M. Bazaga P. (2013). Epigenetic correlates of plant phenotypic plasticity: DNA methylation differs between prickly and nonprickly leaves in heterophyllous Ilex aquifolium (Aquifoliaceae) trees. *Bot. J. Linnean Soc.* 171 441–452. 10.1111/boj.12007

[B61] Hirsch S. Baumberger R. Grossniklaus U. (2013). Epigenetic variation, inheritance, and selection in plant populations. *Cold Spring. Harb. Symp. Quant. Biol.* 77 97–104. 10.1101/sqb.2013.77.014605 23619013

[B62] Huang Y. Pastor W. A. Shen Y. Tahiliani M. Liu D. R. Rao A. (2010). The behaviour of 5-hydroxymethylcytosine in bisulfite sequencing. *PLoS ONE* 5:e8888 10.1371/journal.pone.0008888 PMC2811190 20126651

[B63] Ito H. (2012). Small RNAs and transposon silencing in plants. *Dev. Growth Diff.* 54 100–107. 10.1111/j.1440-169X.2011.01309.x 22150226

[B64] Ito S. D’Alessio A. C. Taranova O. V. Hong K. Sowers L. C. Zhang Y. (2010). Role of Tet proteins in 5mC to 5hmC conversion, ES-cell self-renewal and inner cell mass specification. *Nature* 466 1129–1133. 10.1038/nature09303 20639862 PMC3491567

[B65] Iwasaki M. Paszkowski J. (2014). Identification of genes preventing transgenerational transmission of stress-induced epigenetic states. *Proc. Natl. Acad Sci. U.S.A.* 111 8547–8552. 10.1073/pnas.1402275111 24912148 PMC4060648

[B66] Jacobsen S. E. Meyerowitz E. M. (1997). Hypermethylated SUPERMAN epigenetic alleles in *Arabidopsis*. *Science* 277 1100–1103. 10.1126/science.277.5329.1100 9262479

[B67] Jaligot E. Beule T. Baurens F. C. Billote N. Rival A. (2004). Search for methylation-sensitive amplification polymorphisms associated with the “mantled” variant phenotype in oil palm (*Elaeis guineensis* Jacq.). *Genome* 47 224–228. 10.1139/g03-085 15060619

[B68] Jeon J. Choi J. Lee G.-W. Park S.-Y. Huh A. Dean R. A. (2015). Genome-wide profiling of DNA methylation provides insights into epigenetic regulation of fungal development in a plant pathogenic fungus, *Magnaporthe oryzae*. *Sci. Rep.* 5 8567 10.1038/srep08567 PMC4338423 25708804

[B69] Joyce S. M. Cassells A. C. (2002). Variation in potato microplant morphology in vitro and DNA methylation. *Plant Cell Tissue Organ Cult.* 70 125–137. 10.1023/A:1016312303320

[B70] Jung H. W. Tschaplinski T. J. Wang L. Glazebrook J. Greenberg J. T. (2009). Priming in systemic plant immunity RID D-4021-2009. *Science* 324 89–91. 10.1126/science.1170025 19342588

[B71] Kathiria P. Sidler C. Golubov A. Kalischuk M. Kawchuk L. M. Kovalchuk I. (2010). Tobacco mosaic virus infection results in an increase in recombination frequency and resistance to viral, bacterial, and fungal pathogens in the progeny of infected tobacco plants. *Plant Physiol.* 153 1859–1870. 10.1104/pp.110.157263 20498336 PMC2923882

[B72] Kimatu J. N. Diarso M. Song C. Agboola R. S. Pang J. Qi X. (2011). DNA cytosine methylation alterations associated with aluminium toxicity and low pH in *Sorghum bicolor*. *Afr. J. Agric. Res.* 6 4579–4593. 10.5897/AJAR11.954

[B73] King G. J. Amoah S. Kurup S. (2010). Exploring and exploiting epigenetic variation in crops. *Genome* 53 856–868. 10.1139/G10-059 21076501

[B74] Kinoshita T. Miura A. Choi Y. H. Kinoshita Y. Cao X. F. Jacobsen S. E. (2004). One-way control of FWA imprinting in *Arabidopsis* endosperm by DNA methylation. *Science* 303 521–523. 10.1126/science.1089835 14631047

[B75] Kohler A. Schwindling S. Conrath U. (2002). Benzothiadiazole-induced primingfor potentiated responses to pathogen infection, wounding, and infiltration ofwater into leaves requires the NPR1/NIM1 gene in *Arabidopsis*. *Plant Physiol.* 128 1046–1056. 10.1104/pp.010744 11891259 PMC152216

[B76] Kohler C. Makarevich G. (2006). Epigenetic mechanisms governing seed development in plants. *EMBO Rep.* 7 1223–1227. 10.1038/sj.embor.7400854 17139298 PMC1794698

[B77] Kriaucionis S. Heintz N. (2009). The nuclear DNA base 5-hydroxymethylcytosine is present in Purkinje neurons and the brain. *Science* 324 929–930. 10.1126/science.1169786 19372393 PMC3263819

[B78] Li L. Ming Z. Z. DaiBo L. Jing W. Jian G. MaoJun Z. (2011). Analysis of infection process and methylation-sensitive amplified polymorphism in Zea mays genome stressed by *Rhizoctoniasolani*. *J. Agric. Biotechnol.* 19 243–249.

[B79] Li R.-Q. Huang J.-Z. Zhao H.-J. Fu H.-W. Li Y.-F. Liu G.-Z. (2014). A down-regulated epi-allele of the genomes uncoupled 4 gene generates a xantha marker trait in rice. *Theor. Appl. Genet.* 127 2491–2501. 10.1007/s00122-014-2393-9 25208645

[B80] Li W. Chen W. Qi X. Wang Q. Chen J. (2013). Variation of cytosine methylation in response to water availability in two contrasting growth types of an amphibious plant *Alternantheraphiloxeroides*. *Biochem. Syst. Ecol.* 50 175–181. 10.1016/j.bse.2013.03.053

[B81] Lira-Medeiros C. F. Parisod C. Fernandes R. A. Mata C. S. Cardoso M. A. Ferreira P. C. (2010). Epigenetic variation in mangrove plants occurring in contrasting natural environment. *PLoS ONE* 5:e10326 10.1371/journal.pone.0010326 PMC2859934 20436669

[B82] Long Y Xia W. Li R. Y. Wang J. Shao M. Q. Feng J. King G. J. (2011). Epigentic QTL mapping in *Brassica napus*. *Genetics* 189 1093–10102. 10.1534/genetics.111.131615 21890742 PMC3213370

[B83] Luna E. Bruce T. J. A. Roberts M. R. Flors V. Ton J. (2012). Next generation systemic acquired resistance. *Plant Physiol.* 158 844–853. 10.1104/pp.111.187468 22147520 PMC3271772

[B84] Luo M. Bilodeau P. Koltunow A. Dennis E. S. Peacock W. J. Chaudhury A. M. (1999). Genes controlling fertilization-independent seed development in *Arabidopsis thaliana*. *Proc. Natl Acad. Sci. U.S.A.* 96 296–301. 10.1073/pnas.96.1.296 9874812 PMC15133

[B85] Luykx D. M. A. M. van Ruth S. M. (2008). An overview of analytical methods for determining the geographical origin of food products. *Food Chem.* 107 897–911. 10.1016/j.foodchem.2007.09.038

[B86] Mader E. Ruzicka J. Schmiderer C. Novak J. (2011). Quantitative high-resolution melting analysis for detecting adulterations. *Anal. Biochem.* 409 153–155. 10.1016/j.ab.2010.10.009 20946863

[B87] Madlung A. Comai L. (2004). The effect of stress on genome regulation and structure. *Ann. Bot.* 94 481–495. 10.1093/aob/mch172 15319229 PMC4242226

[B88] Manning K. Tör M. Poole M. Hong Y. Thompson A. J. King G. J. (2006). A naturally occurring epigenetic mutation in a gene encoding an SBP-box transcription factor inhibits tomato fruit ripening. *Nat. Genet.* 38 948–952. 10.1038/ng1841 16832354

[B89] Mason G. Noris E. Lanteri S. Acquadro A. Accotto G. P. Portis E. (2008). Potentiality of methylation-sensitive amplification polymorphism (MSAP) in identifying genes involved in tomato response to tomato yellow leaf curl sardinia virus. *Plant Mol. Biol. Rep.* 26 156–173. 10.1007/s11105-008-0031-x

[B90] Matthes M. Singh R. Cheah S. C. Karp A. (2001). Variation in oil palm (*Elaeis guineensis* Jacq.) tissue culture-derived regenerants revealed by AFLPs with methylation-sensitive enzymes. *Theor. Appl. Genet.* 102 971–979. 10.1007/s001220000491

[B91] Miguel C. Marum L. (2011). An epigenetic view of plant cells cultured in vitro: somaclonal variation and beyond. *J. Exp. Bot.* 62 3713–3725. 10.1093/jxb/err155 21617249

[B92] Miura K. Agetsuma M. Kitano H. Yoshimura A. Matsuoka M. Jacobsen S. E. (2009). A metastable DWARF1 epigenetic mutant affecting plant stature in rice. *Proc. Natl. Acad. Sci. U.S.A.* 106 11218–11223. 10.1073/pnas.0901942106 19541604 PMC2708680

[B93] Molinier J. Ries G. Zipfel C. Hohn B. (2006). Transgeneration memory of stress in plants. *Nature* 442 1046–1049. 10.1038/nature05022 16892047

[B94] Moricová P. Ondøej V. Navrátilová B. Luhová L. (2013). Changes of DNA methylation and hydroxymethylation in plant protoplast cultures. *Acta Biochim. Pol.* 60 33–36. 23505619

[B95] Muenzel M. Globisch D. Carell T. (2011). 5-Hydroxymethylcytosine, the sixth base of the genom. *Angew. Chem. Int. Ed*. 50 6460–6468. 10.1002/anie.201101547 21688365

[B96] Müller K. Hippe J. (1987). “Influence of differences in nutrition on important quality characteristics of some agricultural crops. Plant and Soil Interfaces and Interactions,” in *Developments in Plant and Soil Sciences*, ed. Van Diest A. (Amsterdam: Springer), 28 35–45.

[B97] Naumann U. Daxinger L. Kanno T. Eun C. Long Q. Lorkovic Z. J. (2011). Genetic evidence that DNA methyltransferase DRM2 hasa direct catalytic role in RNA-directed DNA methylation in *Arabidopsis thaliana*. *Genetics* 187 977–979. 10.1534/genetics.110.125401 21212233 PMC3048785

[B98] Nic-Can G. I. López-Torres A. Barredo-Pool F. Wrobel K. Loyola-Vargas V. M. Rojas-Herrera R. (2013). New insights into somatic embryogenesis: LEAFY COTYLEDON 1, BABY BOOM1 and WUSCHEL-RELATED HOMEOBOX4 are epigenetically regulated in *Coffea canephora*. *PLoS ONE* 8:e72160 10.1371/journal.pone.0072160 PMC3748027 23977240

[B99] Ohad N. Margossian L. Hsu Y. C. Williams C. Repetti P. Fischer R. L. (1996). A mutation that allows endosperm development without fertilization. *Proc. Natl Acad. Sci. U.S.A.* 93 5319–5324. 10.1073/pnas.93.11.5319 11607683 PMC39243

[B100] Pan Y. Wang W. Zhao X. Zhu L. Fu B. Li Z. (2011). DNA methylation alterations of rice in response to cold stress. *Plant Omics J.* 4 364–369.

[B101] Pastor V. Luna E. Mauch-Mani B. Ton J. Flors V. (2013). Primed plants do not forget. *Environ. Exp. Bot.* 94 46–56 10.1016/j.envexpbot.2012.02.013

[B102] Plongthongkum N. Diep D. H. Zhang K. (2014). Advances in the profiling of DNA modifications: cytosine methylation and beyond. *Nat. Rev. Genet.* 15 647–661. 10.1038/nrg3772 25159599

[B103] Polaczek P. Kwan K. Campbell J. L. (1998). GATC motifs may alter the conformation of DNA depending on sequence context and N6-adenine methylation status: possible implications for DNA-protein recognition. *Mol. Genet. Genomics* 258 488–493. 10.1007/s004380050759 9669330

[B104] Posner J. L. Baldock J. O. Hedtcke J. L. (2008). Organic and conventional production systems in the wisconsin integrated cropping systems trials: I. Productivity 1990–2002. *Agron. J*. 100 253–260. 10.2134/agrojnl2007.0058

[B105] Quadrana L. Almeida J. Asís R. Duffy R. Guadalupe Dominguez P. Bermúdez L. (2014). Natural occurring epialleles determine vitamin E accumulation in tomato fruits. *Nat. Commun.* 5:3027 10.1038/ncomms5027 24967512

[B106] Quemada H. Roth E. J. Lark K. G. (1987). Changes in methylation of tissue cultured soybean cells detected by digestion with the restriction enzymes HpaII and MspI. *Plant Cell Rep.* 6 63–66. 10.1007/BF00269741 24248452

[B107] Raj S. Bräutigam K. Hamanishi E. T. Wilkins O. Thomasd B. R. Schroeder W. R. Mansfield S. D. (2011). Clone history shapes *Populus* drought responses. *Proc. Natl. Acad. Sci. U.S.A.* 108 12521–12526. 10.1073/pnas.1103341108 21746919 PMC3145742

[B108] Reid L. M. O’Donell C. P. Downell G. (2006). Recent technological advances for the determination of food authenticity. *Trends Food Sci. Technol.* 17 344–353. 10.1016/j.tifs.2006.01.006

[B109] Robertson J. Robertson A. B. Klungland A. (2011a). The presence of 5-hydroxymethylcytosine at the gene promoter and not in the gene body negatively regulates gene expression. *Biochem. Biophys. Res. Commun.* 411 40–43. 10.1016/j.bbrc.2011.06.077 21703242

[B110] Rodríguez López C. M. Guzman Asenjo B. Lloyd A. J. Wilkinson M. (2010a). Direct detection and quantification of methylation in nucleic acid sequences using high-resolution melting analysis. *Anal. Chem.* 82 9100–9108. 10.1021/ac1024057 20945868

[B111] Rodríguez López C. M. Wetten A. C. Wilkinson M. J. (2010b). Progressive erosion of genetic and epigenetic variation in callus-derived cocoa (*Theobroma cacao*) plants. *New Phytol.* 186 56–868. 10.1111/j.1469-8137.2010.03242.x 20406408

[B112] Rodríguez López C. M. Lloyd A. J. Leonard K. Wilkinson M. J. (2012a). Differential effect of three base modifications on DNA thermostability revealed by high resolution melting. *Anal. Chem.* 84 7336–7342. 10.1021/ac301459x 22882125

[B113] Rodríguez López C. M. Morán P. Lago F. Espiñeira M. Beckmann M. Consuegra S. (2012b). Detection and quantification of tissue of origin in salmon and veal products using methylation sensitive AFLPs. *Food Chem.* 131 1493–1498. 10.1016/j.foodchem.2011.09.120

[B114] Rogers J. C. Rogers S. W. (1995). Comparison of the effects of N6-methyldeoxyadenosine and N5-methyldeoxycytosine on transcription from nuclear gene promoters in barley. *Plant J.* 7 221–233. 10.1046/j.1365-313X.1995.7020221.x 7704046

[B115] Rois A. S. Rodríguez López C. M. Cortinhas A. Erben M. Espirito Santo D. Wilkinson M. J. (2013). Epigenetic rather than genetic factors may explain phenotypic divergence between coastal populations of diploid and tetraploid (*Limonium* sp., *Plumbaginaceae)* in Portugal. *BMC Plant Biol*. 13:205–220. 10.1186/1471-2229-13-205 24314092 PMC3884021

[B116] Schellenbaum P. Mohler V. Wenzel G. Walter B. (2008). Variation in DNA methylation patterns of grapevine somaclones (*Vitisvinifera* L.). *BMC Plant Biology.* 8:78–98. 10.1186/1471-2229-8-78 PMC2491626 18627604

[B117] Schmitt F. Oakeley E. J. Jost J. P. (1997). Antibiotics induce genome-wide hypermethylation in cultured *NicotianaTabacum* plants. *J. Biol. Chem.* 272 1534–1540. 10.1074/jbc.272.3.1534 8999825

[B118] Schmitz R. J. He Y. Valdés-López O. Khan S. M. Joshi T. Urich M. A. (2013). Epigenome-wide inheritance of cytosine methylation variants in a recombinant inbred population. *Genome Res.* 23 1663–1674. 10.1101/gr.152538.112 23739894 PMC3787263

[B119] Schulz B. Eckstein R. L. Durka W. (2014). Epigenetic variation reflects dynamic habitat conditions in a rare floodplain herb. *Mol. Ecol.* 23 3523–3537. 10.1111/mec.12835 24943730

[B120] Sentandreu M. A. Sentandreu E. (2011). Peptide biomarkers as a way to determine meat authenticity. *Meat Sci.* 89 280–285. 10.1016/j.meatsci.2011.04.028 21612878

[B121] Sha A. H. Lin X. H. Huang J. B. Zhang D. P. (2005). Analysis of DNA methylation related to rice adult plant resistance to bacterial blight based on methylation-sensitive AFLP (MSAP) analysis. *Mol. Genet. Genomics* 273 484–490. 10.1007/s00438-005-1148-3 15968537

[B122] Shan X. Wang X. Yang G. Wu Y. Su S. Li S. (2013). Analysis of the DNA methylation of maize (*Zea mays* L.) in response to cold stress based on methylation-sensitive amplified polymorphisms. *J. Plant Biol.* 56 32–38. 10.1007/s12374-012-0251-3

[B123] Shiba H. Kakizaki T. Iwano M. Tarutani Y. Watanabe M. Isogai A. (2006). Dominance relationships between self-incompatibility alleles controlled by DNA methylation. *Nat. Genet.* 38 297–299. 10.1038/ng1734 16444272

[B124] Shock L. S. Thakkar P. V. Peterson E. J. Moran R. G. Taylor S. M. (2011). DNA methytransferase 1, cytosine methylation, and cytosine hydroxymethlation in mammalian mitochondria. *Proc. Natl. Acad. Sci. U.S.A.* 108 3630–3635. 10.1073/pnas.1012311108 21321201 PMC3048134

[B125] Slaughter A. Daniel X. Flors V. Luna E. Hohn E. Mauch-Mani B. (2012). Descendants of primed *Arabidopsis* plants exhibit resistance to biotic stress. *Plant Physiol.* 158 835–843. 10.1104/pp.111.191593 22209872 PMC3271771

[B126] Song Y. Ma K. Bo W. Zhang Z. Zhang D. (2012). Sex-specific DNA methylation and gene expression in andromonoecious poplar. *Plant Cell Rep.* 31 1393–1405. 10.1007/s00299-012-1255-7 22476437

[B127] Srancikova A. Horvathova E. Kozics K. (2013). Biological effects of four frequently used medicinal plants of Lamiaceae. *Neoplasma* 60 585–597. 10.4149/neo_2013_076 23906292

[B128] Sternberg N. (1985). Evidence that adenine methylation influences DNA-protein interactions in *Escherichia coli*. *J. Bacteriol.* 164 490–493. 2995323 10.1128/jb.164.1.490-493.1985 PMC214274

[B129] Stokes T. L. Kunkel B. N. Richards E. J. (2002). Epigenetic variation in *Arabidopsis* disease resistance. *Genes Dev.* 16 171–182. 10.1101/gad.952102 11799061 PMC155322

[B130] Stresemann C. Lyko F. (2008). Modes of action of the DNA methyltransferase inhibitors azacytidine and decitabine. *Int. J. Cancer* 123 8–13. 10.1002/ijc.23607 18425818

[B131] Thalhammer A. Hansen A. S. El-Sagheer A. H. Brown T. Schofield C. (2011). Hydroxylation of methylated CpGdinucleotides reverses stabilisation of DNA duplexes by cytosine 5-methylation. *J. Chem. Commun.* 47 5325–5327. 10.1039/c0cc05671e 21451870

[B132] Theiss G. Follmann H. (1980). 5-Methylcytosine formation in wheat embryo DNA. *Biochem. Biophys. Res. Commun.* 94 291–297. 10.1016/S0006-291X(80)80219-1 6770862

[B133] Tiwari S. Schulz R. Ikeda Y. Dytham L. Bravo J. Mathers L. (2008). MATERNALLY EXPRESSED PAB C-TERMINAL, a novel imprinted gene in *Arabidopsis*, encodes the conserved C-terminal domain of polyadenylate binding proteins. *Plant Cell* 20 2387–2398. 10.1105/tpc.108.061929 18796636 PMC2570725

[B134] Tost J. Gut I. G. (2009). “Molecular Techniques for DNA methylation studies,” in *Molecular Diagnostics*, 2nd Edn, eds Patrinos G. Ansorge W. (Amsterdam: Academic Press), 199–228.

[B135] Tricker P. Gibbings G. Rodríguez López C. M. Hadley P. Wilkinson M. J. (2012). Low relative humidity triggers RNA-directed de novo DNA methylation and suppression of genes controlling stomatal development. *J. Exp. Bot.* 63 3799–3813. 10.1093/jxb/ers076 22442411 PMC3733579

[B136] Tricker P. Rodríguez López C. M. Gibbings G. Hadley P. Wilkinson M. J. (2013a). Transgenerational, dynamic methylation of stomata genes in response to low relative humidity. *Int. J. Mol. Sci.* 14 6674–6689. 10.3390/ijms14046674 23531533 PMC3645660

[B137] Tricker P. Rodríguez López C. M. Hadley P. Wagstaff C. Wilkinson M. J. (2013b). Pre-conditioning the epigenetic response to high vapour pressure deficit increases the drought tolerance in *Arabidopsis thaliana*. *Plant Signal. Behav.* 8:e25974 10.4161/psb.25974 PMC4091208 24270688

[B138] Turlings T. C. J. Ton J. (2006). Exploiting scents of distress: the prospect of manipulat-ing herbivore-induced plant odours to enhance the control of agricultural pests. *Curr. Opin. Plant Biol.* 9 421–427. 10.1016/j.pbi.2006.05.010 16723271

[B139] van Leeuwen C. Friant F. Choné X. Tregoat O. Koundouras S. Dubourdieu D. (2004). Influence of climate, aoil, and cultivar on terroir. *Am. J. Enol. Viticul.* 55 207–217.

[B140] Verhoeven K. J. F. Jansen J. J. van Dijk P. J. Biere A. (2010). Stress-induced DNA methylation changes and their heritability in asexual dandelions. *New Phytol.* 185 1108–1118. 10.1111/j.1469-8137.2009.03121.x 20003072

[B141] Waddington C. H. (1942). The epigenotpye. *Endeavour* 1 18–20. 10.1093/ije/dyr184

[B142] Wanunu M. Cohen-Karni D. Johnson R. R. Fields L. Benner J. Peterman N. (2011). Discrimination of methylcytosine from hydroxymethylcytosine in DNA molecules. *J. Am. Chem. Soc.* 133 486–492. 10.1021/ja107836t 21155562 PMC3081508

[B143] Wassenegger M. Heimes S. Riedel L. Sanger H. (1994). RNA-directed de-novo methylation of genomic sequences in plants. *Cell* 76 567–576. 10.1016/0092-8674(94)90119-8 8313476

[B144] Wilusz J. E. Sharp P. A. (2013). A Circuitous Route to Noncoding RNA. *Science 2013*, 340 440–441. 10.1126/science.1238522 PMC4063205 23620042

[B145] Wu H. D’Alessio A. C. Ito S. Wang Z. Cui K. Zhao K. (2011). Genome-wide analysis of 5-hydroxymethylcytosine distribution reveals its dual function in transcriptional regulation in mouse embryonic stem cells. *Genes Dev.* 25 679–684. 10.1101/gad.2036011 21460036 PMC3070931

[B146] Xiong L. Z. Xu C. G. Maroof M. A. S. Zhang Q. F. (1999). Patterns of cytosine methylation in an elite rice hybrid and its parental lines, detected by a methylation sensitive amplification polymorphism technique. *Mol. Gen. Genet.* 261 439–446. 10.1007/s004380050986 10323223

[B147] Yaish M. W. Colasanti J. Rothstein S. J. (2011). The role of epigenetic processes in controlling flowering time in plants exposed to stress. *J. Exp. Bot.* 62 3727–3735. 10.1093/jxb/err177 21633082

[B148] Yu A. Lepere G. Jay F. Wang J. Y. Bapaume L. Wang Y. (2013). Dynamics and biological relevance of DNA demethylation in *Arabidopsis* antibacterial defense. *Proc. Natl. Acad. Sci. U.S.A.* 110 2389–2394. 10.1073/pnas.1211757110 23335630 PMC3568381

[B149] Zhang L. Cheng Z. Qin R. Qiu Y. Wang J.-L. Cui X. (2012). Identification and characterization of an Epi-Allele of FIE1 reveals a regulatory linkage between two epigenetic marks in rice. *Plant Cell* 24 4407–4421. 10.1105/tpc.112.102269 23150632 PMC3531842

[B150] Zhang L. Peng Y. Wei X. Dai Y. Yuan D. Lu Y. (2014). Small RNAs as important regulators for the hybrid vigour of super-hybrid rice. *J. Exp. Bot.* 65 5989–6002. 10.1093/jxb/eru337 25129133 PMC4203131

[B151] Zhang Z. Deng C. Lu Q. Richardson B. (2002). Age-dependent DNA methylation changes in the ITGAL (CD11a) promoter. *Mech. Ageing Dev.* 123 1257–1268. 10.1016/S0047-6374(02)00014-3 12020947

[B152] Zhong S. L. Fei Z. J. Chen Y. R. Zheng Y. Huang M. Y. Vrebalov J. (2013). Single-base resolution methylomes of tomato fruit development reveal epigenome modifications associated with ripening. *Nat. Biotechnol.* 31 154–159. 10.1038/nbt.2462 23354102

[B153] Zufferey V. Murisier F. Schultz H. R. (2000). A model analysis of the photosynthetic response of *Vitis vinifera* L. csv riesling and chasselas leaves in the field: I. interaction of age, light and temperature. *Vitis* 39 19–26.

